# The Nature of Actin-Family Proteins in Chromatin-Modifying Complexes

**DOI:** 10.3389/fgene.2018.00398

**Published:** 2018-09-25

**Authors:** Naeh L. Klages-Mundt, Ashok Kumar, Yuexuan Zhang, Prabodh Kapoor, Xuetong Shen

**Affiliations:** ^1^Science Park Research Division, Department of Epigenetics and Molecular Carcinogenesis, The University of Texas MD Anderson Cancer Center, Houston, TX, United States; ^2^Program in Genetics & Epigenetics, The University of Texas MD Anderson Cancer Center, UTHealth Graduate School of Biomedical Sciences, Houston, TX, United States; ^3^Department of Cellular and Molecular Biology, The University of Texas Health Science Center at Tyler, Tyler, TX, United States; ^4^Key Laboratory of Molecular Biophysics of the Ministry of Education, College of Life Science and Technology, Huazhong University of Science and Technology, Wuhan, China

**Keywords:** nuclear actin, actin-related proteins, chromatin remodeling, INO80 complex, SWR1 complex, SWI/SNF complex, NuA4 complex, DNA repair

## Abstract

Actin is not only one of the most abundant proteins in eukaryotic cells, but also one of the most versatile. In addition to its familiar involvement in enabling contraction and establishing cellular motility and scaffolding in the cytosol, actin has well-documented roles in a variety of processes within the confines of the nucleus, such as transcriptional regulation and DNA repair. Interestingly, monomeric actin as well as actin-related proteins (Arps) are found as stoichiometric subunits of a variety of chromatin remodeling complexes and histone acetyltransferases, raising the question of precisely what roles they serve in these contexts. Actin and Arps are present in unique combinations in chromatin modifiers, helping to establish structural integrity of the complex and enabling a wide range of functions, such as recruiting the complex to nucleosomes to facilitate chromatin remodeling and promoting ATPase activity of the catalytic subunit. Actin and Arps are also thought to help modulate chromatin dynamics and maintain higher-order chromatin structure. Moreover, the presence of actin and Arps in several chromatin modifiers is necessary for promoting genomic integrity and an effective DNA damage response. In this review, we discuss the involvement of actin and Arps in these nuclear complexes that control chromatin remodeling and histone modifications, while also considering avenues for future study to further shed light on their functional importance.

## Introduction

Actin is one of the most fundamental and abundant proteins in eukaryotic cells, comprising up to 20% of total protein mass in certain cell types. Its versatility is arguably unmatched amongst all cellular proteins, given actin’s diverse array of functions in maintaining cellular homeostasis. Until relatively recently, the role of actin in cell physiology was thought to be exclusively relegated to the cytoplasm, primarily functioning in motility, contraction, and scaffolding. However, a newly emerging field has begun to uncover integral roles for actin within the confines of the nucleus. It is now readily apparent that actin and the evolutionarily divergent actin-related proteins are critical to an assortment of nuclear processes, particularly in those modulating various aspects of chromatin dynamics. Herein, we discuss nuclear actin and actin-related proteins, detailing their involvement in nuclear complexes that regulate chromatin remodeling and histone modifications.

## Chromatin Remodeling and Histone Modification

Chromatin structure presents a formidable barrier to proteins and other factors that need to interact with DNA to induce various processes. Thus, accessibility to DNA is largely dependent upon intricate epigenetic control. Chromatin-modifying complexes comprise a crucial class of nuclear enzymatic complexes that affect DNA accessibility, allowing for critical processes such as transcription, replication, and repair. By modulating chromatin structure, chromatin-modifying complexes control the activation and repression of transcription at given regions on the chromosome. This modulation of chromatin structure is generally achieved through applying covalent histone modifications or altering histone composition (Clapier and Cairns, 2009).

Chromatin-modifying complexes can broadly be categorized into chromatin remodelers and histone modifiers. Chromatin remodelers are principally responsible for nucleosome repositioning and reorganization, which is typically achieved in an ATP-dependent manner, as facilitated by the ATPase subunit of the complex (Vignali et al., 2000). Meanwhile, histone modifiers, such as histone acetyltransferases and histone deacetylases, alter the post-translational modification status of histones, adding or removing epigenetic marks that regulate the scope of gene expression (Lee and Workman, 2007). Effectively, both chromatin remodelers and histone modifiers make the chromatin environment either more or less conducive to transcription, while also controlling DNA replication, repair and homologous recombination. Both classes of chromatin-modifying complexes are largely conserved from yeast to mammals (Clapier and Cairns, 2009).

Chromatin remodelers and histone modifiers each share several common features on a structural level. One key structural motif is a helicase-SANT-associated (HSA) domain, which is a large helicase binding module present in a given subunit unique to each complex (Szerlong et al., 2008). For instance, the subunit containing the HSA domain in INO80 is INO80, in SWR1 is SwrI/PieI, in SWI/SNF is Snf2, in RSC is SthI, and in NuA4 is Vid21/Eaf1 (Szerlong et al., 2008; Meagher et al., 2009). Removal of the HSA domain in RSC diminishes ATPase activity *in vitro*, and its presence is necessary for budding yeast viability *in vivo*, demonstrating the physiological significance of this domain (Szerlong et al., 2008). Most subunits in chromatin-modifying complexes, however, are non-enzymatic, instead specialized for a diverse assortment of functions, such as maintaining structural integrity of the complex, recruiting the complex to nucleosomes or other substrates, or aiding in various regulatory mechanisms.

A number of human diseases are associated with defective chromatin remodeling. Various cancers, especially, are strongly correlated with deleterious mutations in chromatin remodeling complexes, particularly SWI/SNF. Indeed, approximately 20% of human cancers possess mutant SWI/SNF subunits (Wilson and Roberts, 2011; Kadoch and Crabtree, 2015). Given this staggeringly high statistic, it seems likely that such mutations may in fact be a direct contributor to carcinogenesis. Defective chromatin modification capability may destabilize the epigenetic status of the genome, disrupting proper gene expression and resulting in elevated cancer predisposition. Indeed, chromatin remodeling complexes may effectively serve as tumor suppressors (Hohmann and Vakoc, 2014).

## Nuclear Actin

Evidence indicative of actin’s presence in the nucleus was initially reported in 1969 (Lane, 1969). This claim remained controversial in the years and decades that followed due to several studies suggesting actin is absent from the nucleus. The actin-binding protein phalloidin does not stain actin filaments inside the nucleus under normal conditions, seemingly indicating that actin filaments are ordinarily absent from the nucleus (Sanger et al., 1980). Moreover, electron microscopy experiments in which canonical actin filaments were not observed in the nucleus bolstered the case negating the presence of nuclear actin. However, numerous studies since 1969 have shown that actin is indeed present in the nucleus in a variety of model eukaryotic organisms. For instance, nuclear actin was detected in hand-isolated *Xenopus* oocyte nuclei using SDS-polyacrylamide gel migration, actin antiserum, DNase I-binding, and comparison of structural characteristics as tools for its identification (Clark and Merriam, 1977). Additionally, treatment with leptomycin B, an inhibitor of nuclear export, results in accumulation of actin in the nucleus (Wada et al., 1998). Moreover, data from *Xenopus* oocytes demonstrate that administration of latrunculin B, a monomeric actin-binding agent that inhibits its polymerization, prevents the actin-mediated nuclear export of RNAs and proteins, implying the presence of actin filaments in the nucleus (Hofmann et al., 2001). Actin has also been discovered in several nuclear complexes, especially ATP-dependent chromatin remodeling complexes and histone acetyltransferases, further supporting the notion that actin is both present and has distinctive roles in the nucleus (Kapoor and Shen, 2014). Further evidence for actin in the nucleus was supported by the fact that numerous proteins that bind filamentous actin or modulate actin dynamics in the cytoplasm – such as cofilin, profilin, and formins – are also present within the nucleus under varying conditions (Castano et al., 2010). The presence of nuclear actin-binding proteins not only provides evidence for actin in the nucleus, but also indicates that its functional state is finely regulated. Over the past several decades, a substantial body of work has been conclusive in defining the presence of actin in the nucleus.

Monoclonal antibodies have been established in mice that are capable of distinguishing actin conformations in the nucleus and cytoplasm, revealing that nuclear actin is conformationally distinct compared to its cytoplasmic counterpart (Gonsior et al., 1999; Schoenenberger et al., 2005). Indeed, several divergent actin species exist in the nucleus, distributed differentially throughout sub-nuclear compartments, as evidenced in experiments using *Arabidopsis* nuclei (Kandasamy et al., 2010). Moreover, nuclear actin conformation also differs across different cell types (Schoenenberger et al., 2005). To further shed light on the nature of actin within the nucleus, a study using fluorescence recovery after photobleaching (FRAP) demonstrated that approximately 20% of the nuclear actin pool is in a dynamic polymeric state, while the remaining 80% is monomeric (McDonald et al., 2006). Interestingly, this polymeric actin pool exists in a non-canonical conformation that prevents phalloidin binding, unlike cytoplasmic F-actin (Hofmann et al., 2001; Krauss et al., 2003; Pendleton et al., 2003; Schoenenberger et al., 2005; Bohnsack et al., 2006; Jockusch et al., 2006; McDonald et al., 2006; Gieni and Hendzel, 2009; Dopie et al., 2012). Taken together, these data reconcile earlier experiments refuting the presence of nuclear actin due to the apparent lack of phalloidin binding in the nucleus under normal conditions.

Nuclear actin filaments have been observed with greater abundance in particular cell types, as well as following certain natural stimuli or non-physiological stress conditions (Grosse and Vartiainen, 2013). For instance, thymidine-induced DNA replication arrest and hydroxyurea-induced replication stress trigger increased actin levels in the nucleus, which is concomitant with a nuclear enrichment of actin regulators (Johnson et al., 2013). Despite filamentous actin being more prevalent in certain cell types, the overall concentration of actin in the nucleus remains fairly constant throughout different cell types and various species (Hofmann, 2009). However, fluctuations in nuclear actin levels and polymerization status can be observed upon various stresses to the cell, including depletion of ATP, DMSO treatment, heat-shock, and administration of actin polymerization inhibitors (Hofmann, 2009). Decreased overall levels of nuclear actin coincide with cells that are in quiescence (Spencer et al., 2011), while increased nuclear actin levels correlate with cells that are differentiating (Xu et al., 2010). Shuttling of free nuclear actin to the cytoplasm diminishes overall actin levels in the nucleus, though incorporation of actin into nuclear protein complexes likely ensures that sufficient levels are sustained in the nucleus (Skarp et al., 2013). As the abundance of actin in the nucleus substantially exceeds the concentration sufficient for spontaneous polymerization (Pollard et al., 2000), the polymerization state of nuclear actin is necessarily regulated in order to maintain the abundant levels of monomeric actin observed. This is perhaps accomplished by various co-factors that localize to the nucleus (Visa and Percipalle, 2010). The levels of monomeric actin in the nucleus are in stark contrast with actin levels observed in the cytoplasm, where actin primarily exists in filaments (Grosse and Vartiainen, 2013). The polymerization state of nuclear actin likely affects its overall function in assorted nuclear metabolic processes.

Actin is now known to be involved in a host of processes associated with nuclear metabolism and chromatin transactions, demonstrating the versatility of this protein in cellular function. Nuclear actin binds transcription factors and is implicated in transcriptional regulation, including gene activation and silencing as well as RNA processing and splicing. In addition, nuclear actin has been shown to bind all three classes of RNA polymerase complexes (Miralles and Visa, 2006). Both polymeric and monomeric nuclear actin are involved in these transcription-related processes. Indeed, actin polymerization inhibitors and actin polymerization-defective mutants have each been found to severely disrupt transcription (McDonald et al., 2006; Ye et al., 2008).

A recent study demonstrated that histone deacetylases (HDACs) 1 and 2 are each regulated by nuclear actin (Serebryannyy et al., 2016), further extending its epigenetic role. Specifically, monomeric actin actively attenuates HDAC function, thereby increasing histone acetylation. Conversely, polymerizing nuclear actin has the opposite effect, promoting HDAC activity and diminishing overall acetylation of histones, likely as a consequence of sequestering monomeric actin species within the nucleus. Thus, it appears that, in addition to its role in promoting certain nuclear processes, nuclear actin may also work to inhibit the activities of certain nuclear protein complexes (Serebryannyy et al., 2016). Perhaps, interaction of actin with HDACs may in turn allow other actin-associated complexes – such as chromatin remodelers and histone acetyltransferases – to relax chromatin into a transcription-ready state.

Nuclear actin is also important for intranuclear transport, as well as nuclear export of proteins and RNA, as demonstrated by studies in *Xenopus* oocytes (Hofmann et al., 2001). Chromosome movement within the nucleus also appears to be facilitated in part by nuclear actin (Chuang et al., 2006; Dundr et al., 2007; Spichal et al., 2016). Nuclear actin also contributes to the maintenance of nuclear structure and the assembly of the nuclear envelope (Krauss et al., 2003). Furthermore, nuclear actin serves as a cofactor in various nuclear signaling pathways (Gieni and Hendzel, 2009). SUMO modifications have also been identified on nuclear actin, serving as a prerequisite for actin localization to the nucleus (Hofmann et al., 2009). Recently, it has been demonstrated that nuclear actin filaments accumulate in daughter cells after cell division, promoting reorganization of chromatin (Baarlink et al., 2017). Abolishing polymerization-competent actin within the nucleus results in increased chromatin compaction in daughter nuclei, which coincides with diminished transcription activity and slower cell growth. These defects in turn coincide with a severe hindrance to early development, as evidenced in experiments with mouse embryos (Baarlink et al., 2017). To ensure proper chromatin reorganization following mitosis, this filamentous actin must be finely regulated. Indeed, cofilin-1 serves as one key regulator in the nucleus, controlling the dynamics of this species of nuclear actin (Baarlink et al., 2017).

In totality, nuclear actin has been implicated in a multitude of distinctive roles within the nucleus, in both filamentous and monomeric forms. These functions ensure proper nuclear metabolism and physiology. As we will discuss next, nuclear actin also plays vital roles in ensuring an effective DNA damage response.

## Nuclear Actin in the Dna Damage Response

DNA is continuously damaged by a variety of endogenous and environmental mutagens, requiring efficient DNA repair pathways to avoid harmful mutations that may eventually lead to genomic instability and cancer. Numerous DNA repair pathways have evolved to deal with the wide array of damage commonly induced throughout the genome. Interestingly, recent research has revealed that nuclear actin plays an important role in DNA repair processes as well, opening a new domain of research in the nuclear actin field (Andrin et al., 2012; Belin et al., 2015; Spichal et al., 2016; Caridi et al., 2018; Schrank et al., 2018). Actin filament formation is induced in the nucleus following DNA double-strand breaks and is thought to have crucial roles in promoting their effective repair (Andrin et al., 2012; Sun et al., 2017; Caridi et al., 2018; Schrank et al., 2018). Moreover, many repair factors such as the nuclease Mre11, recombinase Rad51, and non-homologous end-joining heterodimer Ku70-Ku80 associate with polymerized actin *in vitro*, and are suggested to help recruit or stabilize these repair factors at the damage site (Andrin et al., 2012). Consistently, disruption of actin polymerization inhibits double-strand break repair *in vivo* – and inhibits non-homologous end-joining in particular *in vitro*, suggesting a prominent role of polymerized nuclear actin in DNA damage repair. Efficient repair of double-strand breaks through homologous recombination requires nuclear actin structures as well (Spichal et al., 2016; Caridi et al., 2018; Schrank et al., 2018). Indeed, overexpression of a nuclear localized actin variant defective in filament formation resulted in a significant decrease in homologous recombination efficiency (Schrank et al., 2018). Other DNA damage response proteins, including p53 and JMY, also interact with actin filaments following DNA damage, providing further evidence for a significant role of nuclear actin in regulating DNA repair (Metcalfe et al., 1999; Lin et al., 2014).

Although the detailed mechanism underlying nuclear actin filaments in DNA repair is lacking, various actin-binding proteins and regulators are known to control actin’s polymerization in response to DNA damage. The actin nucleation factors Formin and Spire-1/Spire-2 are specifically linked to double-strand break repair (Belin et al., 2015). Moreover, several actin-binding proteins have been shown to interact with DNA damage response proteins. Filamin-A, for instance, interacts with the homologous recombination protein BRCA2, and depletion of Filamin-A leads to sensitivity to DNA damaging agents (Yuan and Shen, 2001). Additionally, overexpression of cofilin, an actin depolymerization factor, sensitizes cells to radiation-induced DNA damage, which further supports the prominent role of polymerized actin in DNA repair processes (Lee et al., 2005). Taken together, these studies suggest that nuclear actin – probably in its polymeric state – plays a significant role in several DNA repair processes.

As we will discuss later in this chapter, monomeric nuclear actin in the context of chromatin-modifying complexes also plays pivotal roles in promoting DNA repair and transcription. Intriguingly, such actin monomers are incorporated into several chromatin-remodelers and histone modifiers (Olave et al., 2002; Chen and Shen, 2007; Meagher et al., 2007; Kapoor and Shen, 2014), though its precise roles in these complexes have only recently begun to be elucidated. In the following sections of this review, we will focus on the current understanding of the functional roles of actin and actin-related proteins in the context of chromatin remodeling complexes and histone acetyltransferases.

## Functions of Nuclear Actin-Related Proteins (Arps 4–9)

Actin-related proteins (Arps) are proteins that exhibit structural and functional similarities to conventional actin. Found in all eukaryotic cells, Arps are thought to have evolved from actin through a series of gene duplication events prior to the diversification of eukaryotic kingdoms (Meagher et al., 2009). Each member of the actin family, including Arps, possesses a core actin fold domain that has a highly conserved tertiary structure (Kabsch and Holmes, 1995; Fenn et al., 2011; Gerhold et al., 2012). Each Arp and conventional actin also shares a conserved ATP/ADP-binding motif that enables the protein’s ATPase activity (Muller et al., 2005). While still possessing the same overall basal structure, the surface structure of each protein is varied throughout the actin and Arp family (Mullins et al., 1996). Ten subfamilies of Arps have been characterized in eukaryotes, Arp1–Arp10, arranged by the extent of their similarity with actin (Poch and Winsor, 1997). While Arp1 is most conserved with conventional actin, Arp10 is the most divergent. Furthermore, while Arp subfamilies 1–3 and 10 are typically found in the cytoplasm, Arps 4–9 localize to the nucleus, where they play vital roles in a variety of nuclear metabolic processes (Harata et al., 2000). Arps perform distinctive functions related to conventional actin; however, because of their surface structure variation, they may perform more specific tasks relative to the more general functions coordinated by actin. Consistently, expression of some Arps is largely tissue-specific, likely regulating cell type-specific processes like gene expression, development, or differentiation (Harata et al., 1999a; Heid et al., 2002; Kuroda et al., 2002; Lessard et al., 2007; Wu et al., 2007; Hara et al., 2008; Yoo et al., 2009; Dion et al., 2010; Krasteva et al., 2012; Morita and Hayashi, 2014; Qin et al., 2014). Nuclear Arps appear to be essential for cellular function in animals, as naturally defective alleles have not yet been observed. Strikingly, however, Arp mutants have been identified in *Arabidopsis*, suggesting that plants better tolerate Arp-deficiency than do animals (Meagher et al., 2010).

Unlike canonical actin, Arps do not polymerize into long filaments, instead residing as monomers in the nucleus, as revealed by the crystal and solution structures of Arp4 and Arp8 (Fenn et al., 2011). Throughout evolution, insertions and deletions in these Arp subfamilies have resulted in a variety of features distinctive to each Arp. Though each individual Arp contains diverse structural features, they are unified by their propensity to be components of various protein complexes, wherein they contribute to an assortment of nuclear or cytosolic functions. Amongst the nuclear functions of Arps, perhaps the most notable include nucleosome recognition, transcriptional regulation, and higher-level chromatin organization (Meagher et al., 2009; Dion et al., 2010; Oma and Harata, 2011).

Though typically localized to the cytoplasm, both Arp2 and Arp3 can also shuttle to the nucleus, where, as components of the Arp2/3 actin nucleation complex, they have defined roles in transcriptional regulation and actin polymerization dynamics (Yoo et al., 2007). Recently, it was demonstrated in *Xenopus* egg extracts that Mre11 recruits the Arp2/3 complex to DNA double-strand break sites (Schrank et al., 2018). In human U2OS cells, WASP (an Arp2/3 activating protein and regulator) specifically activates Arp2/3 at double-strand breaks designated for homologous recombination in G2 of the cell cycle, where it then promotes the assembly of nuclear actin filaments. These events in turn promote DNA end resection and progression of homologous recombination, as well as single-strand annealing (though, in this assay, do not appear to be involved in regulating non-homologous end-joining or microhomology-mediated end-joining) (Schrank et al., 2018). Arp2/3-mediated action nucleation also enables homologous recombination at double-strand breaks in heterochromatin (Caridi et al., 2018). Double-strand break repair foci are re-directed to the nuclear periphery by nuclear myosin along the Arp2/3-mediated filamentous actin to prevent ectopic recombination in the heterochromatin region (Caridi et al., 2018). Depletion or chemical inhibition of Arp2/3 components, WASP, or myosin results in genomic instability and defective homologous recombination and single-strand annealing, underscoring the critical roles Arp2 and Arp3 play when present within the nucleus (Caridi et al., 2018; Schrank et al., 2018). These findings in human cell lines are consistent with studies in budding yeast demonstrating that co-depletion of individual Arp2/3 complex components and Mre11 or Sgs1 results in synthetic lethality (van Pel et al., 2013).

The most conserved amongst nuclear Arps is Arp4. *Arp4*, which is an essential gene in yeast (Harata et al., 1994), is also the most commonly found Arp in nuclear protein complexes, wherein it plays a prominent role in regulating gene expression and transcription (Meagher et al., 2009; Dion et al., 2010; Oma and Harata, 2011), interacting with several complexes involved in DNA transactions, including histone deacetylase 2 (Joshi et al., 2013). Arp4 is also known to exist independent of inclusion in nuclear complexes, perhaps possessing distinct or ancillary roles in this context (Sunada et al., 2005). Both *in vitro* and *in vivo*, Arp4 and Arp8 have been found to bind core histones, potentially suggesting a role in controlling chromatin structure (Harata et al., 1999b; Galarneau et al., 2000; Shen et al., 2003; Downs et al., 2004; Gerhold et al., 2012; Saravanan et al., 2012). In support of this notion, the large loop insertions in Arp4 (as well as in Arp8) contain high percentages of acidic amino acid residues, which are thought to facilitate interactions with histone proteins, which are mostly basic (Fenn et al., 2011). By binding core histones and histone side-chains, both Arp4 and Arp8 may assist recruitment of various complexes to chromatin, thus regulating remodeling and transcription (Harata et al., 1999b, 2002; Galarneau et al., 2000; Shen et al., 2003; Downs et al., 2004; Gerhold et al., 2012; Saravanan et al., 2012). In budding yeast, Arp4 has also been shown to bind the linker histone Hho1, a unit key in establishing and organizing higher-order chromatin structure (Georgieva et al., 2015). An Arp4 mutant strain (G187R), which displays only minimal morphological defects, exhibits substantial cellular and nuclear morphological deviation and a dramatic collapse of higher-order chromatin structure upon simultaneous knockout of *HHO1* (Georgieva et al., 2015). Indeed, this *Arp4*-*HHO1* double mutant disrupts chromatin loop organization, weakens chromatin compaction, and exhibits excessively swollen nuclei (Georgieva et al., 2015). Moreover, as Arp4 inhibits actin filament assembly *in vitro*, it may serve as a factor regulating the monomeric state of nuclear actin overall (Fenn et al., 2011). Arp4 also appears to play an important role in proper G2/M cell cycle checkpoint progression, likely by ensuring complete kinetochore assembly and cohesin binding (Ogiwara et al., 2007b; van Attikum et al., 2007). Arp4 appears to be the most versatile in the nuclear Arp family, assisting and regulating numerous nuclear metabolic processes.

Arps 5–9, which are more evolutionarily and structurally divergent than Arp4 relative to actin, are also multifaceted in their array of functions within the nucleus. Arp5 has been shown to play key roles in smooth muscle cells, wherein it binds to and negatively regulates myocardin, perhaps in an INO80-dependent manner (Morita and Hayashi, 2014). Arp6 has been shown to interact with centromeres, certain gene promoters, and telomeres, suggesting possible roles in transcription or replication regulation (Yoshida et al., 2010). Indeed, in animals and fission yeast, Arp6 is believed to be involved in silencing at telomeres and in heterochromatin regions (Kato et al., 2001; Ueno et al., 2004; Ohfuchi et al., 2006). Furthermore, through centromere binding, Arp6 assists the process by which chromatin anchors to the nuclear periphery (Yoshida et al., 2010). Arp6 is also necessary for maintaining microtubule integrity and DNase activity in yeast (Luo et al., 2015). Arp6 also contributes to nucleolus function and the regulation of rDNA transcription, at least partially in a SWR1-dependent manner (Kitamura et al., 2015). A study using chicken DT40 cells found that Arp6 and histone variant H2A.Z help facilitate spatial positioning of chromatin and may be implicated in normal development (Maruyama et al., 2012). By regulating gene expression in *Arabidopsis*, Arp6 has also been shown to have a significant impact on regulating plant development (Deal et al., 2005, 2007; Meagher et al., 2010). In *Arabidopsis*, Arp6 is also responsible for depositing H2A.Z at genes involved in responding to phosphate starvation (Smith et al., 2010). Arp7 has been shown to regulate *Arabidopsis* embryogenesis and development (Kandasamy et al., 2005). Arp8, which is recruited to chromosomes during mitosis, may be either directly or indirectly involved in ensuring proper mitotic chromosome alignment and chromosomal segregation (Aoyama et al., 2008). Arp9, the least conserved nuclear Arp, plays a necessary role in fruiting initiation in certain plants (Nakazawa et al., 2016). As will be discussed in the following sections, in the context of chromatin-modifying complexes (and perhaps independently as well), several Arps play key roles in modulating chromatin dynamics, while also facilitating DNA repair and maintaining genomic stability.

## Mechanisms of Nuclear Actin and Arps in Chromatin-Modifying Complexes

**FIGURE 1:**
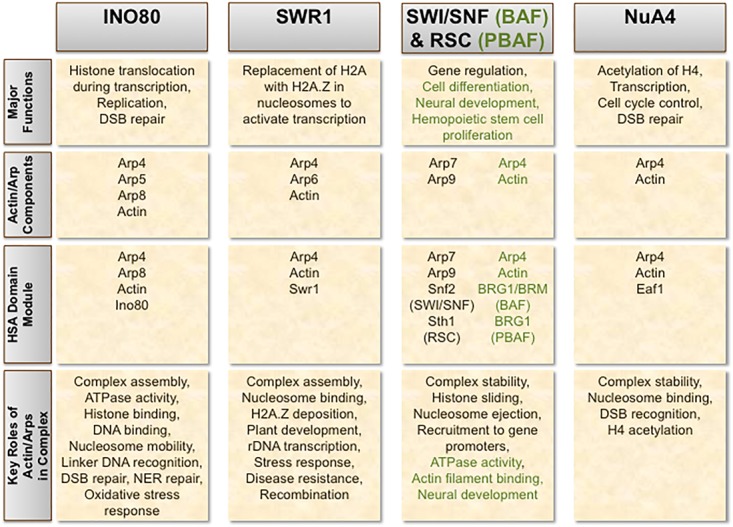
Incorporation of actin and Arps in chromatin-modifying complexes. The chromatin remodeling complexes INO80, SWR1, SWI/SNF, and RSC, as well as the histone acetyltransferase NuA4, each contain a combination of actin and/or actin-related proteins (Arps) as important stoichiometric subunits. The respective human orthologs of SWI/SNF and RSC – BAF and PBAF – exhibit evolutionary divergence from their counterparts in yeast. Structural and functional differences in these human complexes compared to SWI/SNF and RSC are denoted in green.

Actin and Arps are key components in several chromatin-modifying complexes across a wide range of organisms, wherein they serve critical roles in establishing proper complex assembly and modulating chromatin dynamics and structure (Boyer and Peterson, 2000; Olave et al., 2002; Chen and Shen, 2007; Oma and Harata, 2011; Kapoor and Shen, 2014). Distinctive combinations of actin and Arps have been characterized in ATP-dependent chromatin remodelers such as INO80 (Shen et al., 2000; Fenn et al., 2011; Kapoor et al., 2013; Tosi et al., 2013), SWR1 (Krogan et al., 2003; Mizuguchi et al., 2004), SWI/SNF (Cairns et al., 1998; Peterson et al., 1998; Schubert et al., 2013), BAF (Zhao et al., 1998), and RSC (Cairns et al., 1998; Szerlong et al., 2003), as well as some histone acetyltransferases including NuA4/TIP60 (Galarneau et al., 2000; Ikura et al., 2000) (**Figure 1**). Complexes that integrate both remodeling and acetyltransferase activity, such as p400 in humans, also possess actin and Arp subunits (Fuchs et al., 2001). Actin is universally monomeric in each complex in which it is present, paired in unique combinations with particular nuclear Arps. At least in the case of INO80, Arps are thought to maintain actin’s monomeric state within the complex (Fenn et al., 2011). This regulatory function of Arps might be prevalent amongst other actin-containing chromatin-modifying complexes as well.

While detailed mechanisms are still being elucidated for Arps in chromatin-modifying complexes, genetic and biochemical approaches have recently shed some light on the functional role of actin-family proteins in these complexes. In particular, chromatin-modifying complexes require Arps to perform their key roles in maintaining epigenetic control (Meagher et al., 2010). The presence of actin and Arps is critical for assembly of chromatin-modifying complexes, as well as for maintaining their structural integrity. Actin and Arps are also required for ATP-dependent chromatin remodeling in several complexes, regulating the ATPase domain (Shen et al., 2003). Yeast presents an ideal model system for investigating the role of nuclear actin in these complexes, as only a single actin-encoding gene, *ACT1*, is present in the yeast genome, thereby clarifying the experimental readout and interpretation of such studies (Kapoor and Shen, 2014).

When present in SWI/SNF and RSC, Arps may form heterodimers with an Arp counterpart (Arp7–Arp9), together promoting structural integrity of the complex (Szerlong et al., 2003; Schubert et al., 2013). This Arp-Arp heterodimerization likely exemplifies another evolutionarily conserved feature amongst these classes of nuclear complexes (Szerlong et al., 2003; Clapier and Cairns, 2009). In other chromatin-modifying complexes, including INO80, SWR1/SRCAP, BAF, PBAF, and NuA4/TIP60, actin and Arp4 pair with each other. This actin-Arp4 pair forms a sub-module with the HSA domain of the catalytic subunit unique to each complex. The structural arrangement of this actin-Arp4 conjugate is similar to that of the Arp7–Arp9 pair found in SWI/SNF and RSC (Schubert et al., 2013; Cao et al., 2016). The overall similarities in structure and function between these conjugates in chromatin-modifying complexes strongly suggest they evolved from a common ancestor (Cao et al., 2016). As Arp4 is the most conserved nuclear Arp, it seems fitting that it is the most commonly found Arp in chromatin-modifying complexes. As such, the actin-Arp4 module might be a fundamental structural and functional component of actin-family proteins, allowing combinatorial formation of additional Arp subunits in these complexes. Co-immunoprecipitation assays have found Arp5 to associate with itself as well, although the physiological significance of this interaction remains unclear (Morita and Hayashi, 2014).

The HSA domain serves as a binding platform not just for actin-Arp4 conjugates in chromatin-modifying complexes, but also for Arp-Arp conjugates as well, as is the case in the chromatin remodelers SWI/SNF and RSC (Szerlong et al., 2008). The HSA domain effectively targets actin-Arp and Arp-Arp pairs to the complex, usually through interactions with hydrophobic residues on the HSA domain of the catalytic subunit (Szerlong et al., 2008; Cao et al., 2016). When the HSA domain is precluded from chromatin remodelers, Arps are consequently no longer incorporated into the complex, further highlighting this domain’s importance in ensuring complete complex assembly (Szerlong et al., 2008). Consistently, chromatin-modifying complexes that lack an HSA domain in any of its subunits have no apparent interactions with nuclear actin or Arps (Szerlong et al., 2008). When bound to actin-Arp or Arp-Arp heterodimers, the HSA domain sub-complex may potentially promote further associations with other complex subunits. Furthermore, the structure of the HSA domain module provides an additional means of inhibiting polymerization of its actin/Arp subunits in several remodeling complexes (Fenn et al., 2011; Kapoor et al., 2013; Lobsiger et al., 2014; Cao et al., 2016).

As discussed earlier, Arps may also contribute to chromatin state regulation independently of their physical involvement in chromatin-modifying complexes, as several Arps directly interact with histones (Harata et al., 1999b; Galarneau et al., 2000; Shen et al., 2003; Downs et al., 2004; Gerhold et al., 2012; Saravanan et al., 2012). Perhaps free, non-complex-associated Arps may assist in targeting chromatin-modifying complexes to nucleosomes. Conceivably, nuclear actin and/or Arps may also facilitate binding of chromatin-modifying complexes to filamentous nuclear actin, perhaps constituting a means of mobility around the nucleus. Clearly delineating the complex-dependent versus independent functions of nuclear Arps should be an important consideration in future studies.

## INO80

INO80 is an evolutionarily conserved ATP-dependent chromatin remodeling complex that is responsible for histone translocation during transcription, replication, and DNA double-strand break repair (Morrison et al., 2004; van Attikum et al., 2004; Bao and Shen, 2007; Kawashima et al., 2007; Papamichos-Chronakis and Peterson, 2008; Shimada et al., 2008; Clapier and Cairns, 2009; Conaway and Conaway, 2009; Morrison and Shen, 2009). The structure and topology of the INO80 complex reveal it to be distinct from other remodeling complexes both in structure and by mechanism. Rather than possessing a cavity specialized for nucleosome binding, INO80 instead adopts a conformation that flexibly cradles nucleosome substrates (Tosi et al., 2013). The INO80 complex contains Arp4, Arp5, Arp8, and an actin monomer as stoichiometric subunits (Shen et al., 2000; Fenn et al., 2011; Kapoor et al., 2013; Tosi et al., 2013). Incorporation of each Arp and actin subunit in INO80 is evolutionarily conserved between yeast and humans, illustrating the integral roles they play in the complex’s function (Jin et al., 2005). Arp5 and Arp8 are each apparently specific to INO80 – and thus likely contribute to distinctive processes – whereas Arp4 is also present in other chromatin-modifying complexes. Of these subunits, actin, Arp4, and Arp8 form a sub-module with the HSA domain of the INO80 DNA-dependent ATPase to constitute the core of the INO80 complex (Szerlong et al., 2008). *In vitro* experiments demonstrate that this sub-module is required for INO80 to interact with chromatin, greatly increasing affinity to DNA relative to each individual subunit’s DNA affinity (Gerhold et al., 2012).

INO80-dependent chromatin remodeling depends on the presence of the monomeric actin subunit in the complex (Kapoor et al., 2013). The actin monomer in INO80 does not have its barbed end exposed, thus preventing nucleation of actin filaments (Fenn et al., 2011; Kapoor et al., 2013). Arp4 and Arp8 effectively cap the barbed end of actin in solution, stabilizing the monomeric form and inhibiting its polymerization, while retaining the actin subunit in the complex. This leaves only actin’s pointed end free to interact with substrates such as chromatin (Fenn et al., 2011; Kapoor et al., 2013). The pointed end of actin in INO80 has a critical role in recognizing linker DNA between nucleosomes, as evidenced by an A58T missense mutation in subdomain 2 of the pointed end of actin in budding yeast hindering INO80’s ability to bind extranucleosomal DNA (Kapoor et al., 2013). Other than impaired INO80 binding to nucleosomes, this mutation also diminishes the complex’s ATPase activity (Kapoor et al., 2013). It has been suggested that actin may work in conjunction with Arp4 in the INO80 sub-module to facilitate this interaction with extranucleosomal DNA, an interesting prospect given that the actin-Arp4 association is conserved amongst several chromatin-modifying complexes and this interaction with linker DNA might therefore be applicable to other complexes as well (Bartholomew, 2013). In binding linker DNA, INO80 assists in uniformly spacing nucleosomes (Udugama et al., 2011). While the overall significance of INO80 binding with linker DNA is still not entirely clear, this interaction may also be involved in fostering proper chromatin architecture and higher-order chromatin structure (Varga-Weisz and Becker, 2006; Georgieva et al., 2015).

Combined genetic and biochemical analyses have uncovered details on the practical importance of actin and Arps in INO80 and for maintaining a healthy physiological state. In budding yeast, both *Arp4* and *ACT1* are essential genes, as their depletion leads to loss of viability (Shen et al., 2000). Arp4 has been shown to recognize core histones in both *in vitro* and *in vivo* experiments, preferentially binding to histone H2A, H2B, and H3. It is possible that, when subunits of INO80, they may promote recruitment of the complex to nucleosomes (Harata et al., 1999b; Galarneau et al., 2000). Meanwhile, while Arp5 is not essential for viability, its depletion leads to defective INO80 function in budding yeast (Shen et al., 2003). Moreover, *in vitro* ATPase activity, DNA binding, and nucleosome mobility are each hindered as a result of Arp5 depletion in yeast, with similar results reported following Arp8 depletion (Shen et al., 2003). Arp8 also seems to be indispensable for complete INO80 complex assembly, as Arp8-depleted cells lack Arp4 and actin in INO80. Contrarily, depletion of Arp4 or actin does not affect INO80 complex formation (Shen et al., 2003). Thus, it appears that Arp8 plays a unique structural role in proper assembly of the INO80 complex. In INO80, Arp8 has several ancillary roles as well, including facilitating sister chromatid cohesion (Ogiwara et al., 2007a) and controlling the distribution of unacetylated histone variant H2A.Z throughout the genome (Papamichos-Chronakis et al., 2011). Similar to Arp4, Arp8 also binds core histones, providing another possible mechanism for recruitment of INO80 to nucleosomes. While capable of binding each of the four core histones comprising the nucleosome, Arp8 exhibits a particularly strong affinity toward H3–H4 tetramers (Shen et al., 2003; Gerhold et al., 2012; Saravanan et al., 2012). According to one study, however, dimerization of Arp8 is required to stabilize this interaction with H3–H4 tetramers (Saravanan et al., 2012). Given that stoichiometric analysis of INO80 reveals only one Arp8 protein present in the complex (Kapoor et al., 2013), further research is needed to determine the precise role Arp8 may play in histone and nucleosome recognition in the context of the INO80 complex. One possibility is that the actin-Arp4-Arp8-HSA domain sub-module of INO80 binds H3–H4 tetramers more efficiently than monomeric Arp8 itself. Indeed, *in vitro* experiments indicate that this sub-module binds both H3–H4 tetramers and nucleosomes with greater affinity than does monomeric Arp8, according to the dissociation constants calculated in titration assays (Gerhold et al., 2012).

**FIGURE 2:**
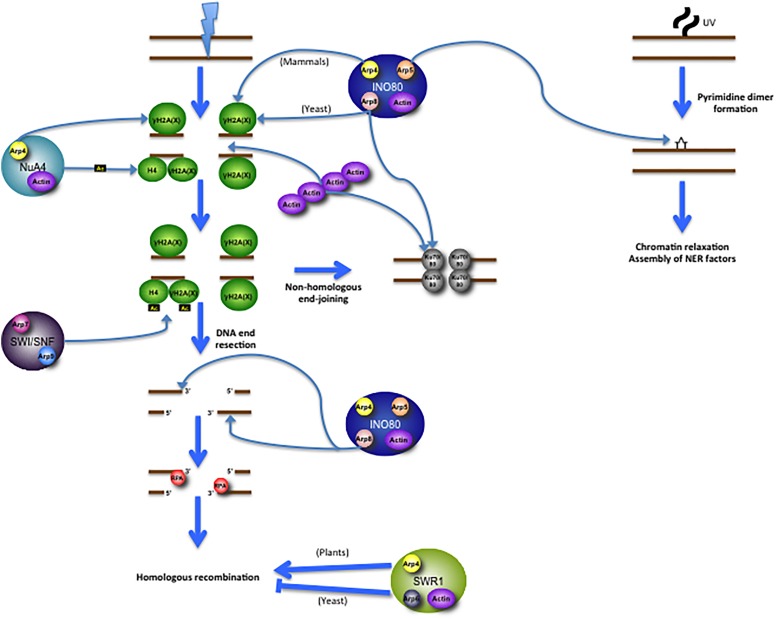
Multifaceted roles for actin/Arp subunits of chromatin-modifying complexes in promoting DNA repair. Following a DNA double-strand break (DSB), INO80 is recruited to γH2A (in yeast; γH2AX in mammals) via its Arp subunits (Arp8 in yeast and Arp4 in mammals). NuA4 is recruited to γH2A via its Arp4 subunit, after which it then acetylates both γH2A and H4. This acetylation event promotes recruitment of SWI/SNF, which then promotes the further propagation of H2A/H2AX phosphorylation. The Arp8 subunit of INO80 promotes DNA resection, binds to the 3′-overhangs, and enables RPA foci, which then get loaded onto the 3′-overhangs. RPA is then replaced by the recombinase RAD51, allowing for homologous recombination. SWR1 is thought to have contrasting roles in the promotion of recombination; in plants Arp subunits directly promote recombination, while in yeast Arp subunits apparently diminish recombination capability. INO80 also has roles in non-homologous end-joining, wherein its Arp8 subunit promotes Ku80 binding to the DNA ends, where it interacts with Ku70 to form a heterodimer. Polymeric actin is also required for Ku80 recruitment to the DSB, though actin filaments promote homologous recombination as well, perhaps in an independently regulated process. INO80 is also involved in the repair of UV-induced photo lesions. Following UV damage, its Arp5 and INO80 subunits are recruited to the photo lesion (indicated here as a pyrimidine dimer), suggesting that the whole complex is likely present at the damage site. These subunits directly promote chromatin relaxation and the assembly of nucleotide excision repair proteins.

The actin and Arp subunits of INO80 have been demonstrated to promote the remodeling complex’s response to DNA damage as well as to various cellular stresses in yeast, plants, and mammalian cells (**Figure 2**). Budding yeast containing an A58T missense mutation in actin subdomain 2 exhibit hypersensitivity to hydroxyurea (Kapoor et al., 2013), an agent that depletes the dNTP pool, stalling replication forks and potentially leading to double-strand breaks following replication fork collapse. Additionally, yeast cells deleted in INO80 subunits Arp5, Arp8 or INO80 are hypersensitive to hydroxyurea and the DNA alkylating agent methyl methanesulfonate (MMS) (van Attikum et al., 2004). These results may in part be attributed to INO80’s role in promoting replication fork progression. The INO80 and Arp5 subunits bind to DNA replication origins, where, upon replication fork stalling after hydroxyurea treatment, they promote its recovery (Shimada et al., 2008). Plants lacking functional Arp5 also exhibit hypersensitivity to double-strand break-inducing agents (Kandasamy et al., 2009). *Arp8* knockout also renders human cells hypersensitive to double-strand break-inducing agents and replication inhibitors (Osakabe et al., 2014). Depletion of Arp5 in human cells reduces γH2AX foci following bleomycin treatment, further suggesting a role for Arp5 in the double-strand break response; inversely, γH2AX levels increase when *Arp5* is overexpressed (Kitayama et al., 2009). In mammalian cells, the Arp8 subunit specifically recruits INO80 to γH2AX foci, thus serving as a means for recognition and accession of DNA damage sites (Kashiwaba et al., 2010). In budding yeast, however, recruitment of INO80 to DNA damage sites is dependent on Arp4 or Nhp10 rather than Arp8, indicating an evolutionary divergence in subunit function (Downs et al., 2004; Morrison et al., 2004; Kashiwaba et al., 2010). Three INO80 subunits – Arp5, Arp8, and INO80 – each bind near the double-strand break sites, suggesting the full complex itself is likely recruited to the lesion. Moreover, Arp8 is required to process double-strand breaks into single-stranded DNA intermediates (van Attikum et al., 2004). Arp8 is capable of binding both double-stranded DNA and single-stranded DNA (preferentially binding to the latter) in human cells, an event regulated by the presence of ATP (Osakabe et al., 2014). Arp8 also binds 3′-overhangs which, given their formation during DNA end resection, may help facilitate damage recognition or process recombination intermediates (Osakabe et al., 2014). This notion is consistent with a prior report showing that Arp8 knockdown abrogates formation of RPA foci (Gospodinov et al., 2011). During the repair process, Arp8 also plays a role in sister chromatid and interchromosomal recombination (Kawashima et al., 2007; Horigome et al., 2014). Furthermore, *Arp8* mutations prevent several repair proteins from binding at a double-strand break site, including Mre11, Mec1 Ku80, and RPA, leading to defective end resection and G2/M checkpoint activation (van Attikum et al., 2007; Gospodinov et al., 2011). As Ku80 retention at double-strand break sites is also purportedly dependent on polymeric actin (Andrin et al., 2012), it is conceivable that INO80 (or its Arp subunits, independently) may work together with actin filaments to promote efficient double-strand break repair. In addition to their role in enabling double-strand break repair, the INO80 and Arp5 subunits are each recruited to UV-induced photo lesions, where they promote chromatin relaxation and the assembly of nucleotide excision repair proteins to enable repair (Jiang et al., 2010). Studies of INO80 in DNA repair have thus revealed that its Arp subunits play integral roles in facilitating double-strand break repair pathways and nucleotide excision repair (**Figure 2**).

Recently, INO80 was found to help mitigate oxidative stress by binding to regulatory sites and inducing the expression of the heme oxygenase-encoding gene *HMOX1* (Takahashi et al., 2017). Expression of *HMOX1* following oxidative stress was severely reduced in Arp5 and Arp8 knockout cells. Interestingly, their results using these knockouts suggest independent roles for Arp5 and Arp8 in promoting *HMOX1* expression. Only in the Arp8 knockout was INO80 unable to efficiently bind chromatin, suggesting that Arp8 may recruit the complex to the regulatory site. Meanwhile, the Arp5 subunit promotes INO80-mediated remodeling of the surrounding nucleosomes following oxidative stress. The presence of Arp5 also enables the transcriptional activator Nrf2 to bind to the regulatory sites of *HMOX1*, thereby promoting its expression (Takahashi et al., 2017). This study reveals an additional function for Arps in responding to cellular stresses in an INO80-dependent manner.

Taken together, the actin and Arp subunits of INO80 play key roles in mediating the complex’s response to DNA damage and oxidative stress. Given that oxidative stress notably induces reactive oxygen species-mediated base damage on the DNA (amongst other lesions), INO80 may play a role in either responding to or mitigating oxidative DNA damage in addition to its better-defined roles in double-strand break repair. Perhaps future studies will implicate INO80 in an even wider array of pathways or stress responses that are actin- and/or Arp-dependent.

## SWR1 (SRCAP)

SWR1 is another ATP-dependent chromatin remodeling complex, principally responsible for transcriptional regulation. By depositing H2A.Z histone variants within nucleosomes at specific locations on the chromatin, replacing the canonical histone H2A subunit, SWR1 effectively reconstructs the nucleosome structure. This process helps activate the transcription state (Krogan et al., 2003; Mizuguchi et al., 2004; Ruhl et al., 2006; Wong et al., 2007; Clapier and Cairns, 2009). Actin, Arp4, and Arp6 are each present in the SWR1 complex (Krogan et al., 2003; Mizuguchi et al., 2004). Together with the catalytic DNA-dependent ATPase Swr1, these subunits form the core sub-complex of SWR1. Just as in the case of INO80, actin and Arp4 associate with the HSA domain of the catalytic subunit (Szerlong et al., 2008). The crystal structure of actin in complex with Arp4 and the Swr1 HSA domain was recently published, providing a detailed understanding of how the actin subunit is maintained in its monomeric state (Cao et al., 2016). While the C-terminus of the HSA domain of Swr1 binds a series of hydrophobic residues on actin, the N-terminus binds Arp4, which in turn induces twists in actin that transform actin’s pointed end, impeding its polymerization potential (Cao et al., 2016). SWR1 exhibits high structural and functional similarity with the human chromatin remodeling complex SRCAP (Ruhl et al., 2006; Wong et al., 2007).

Both Arp4 and Arp6 contribute to several crucial processes in SWR1. Their presence enables the complete assembly of individual subunits into the SWR1 complex (Wu et al., 2005, 2009). In addition, Arp4 and Arp6 enable SWR1 to associate with nucleosomes, and Arp4 is required for SWR1-mediated H2A.Z deposition *in vitro* (Wu et al., 2005, 2009). As discussed earlier, Arp4’s ability to recognize and bind core histones may be mechanistically relevant to chromatin-modifying complexes (Harata et al., 1999b; Galarneau et al., 2000), including SWR1, by recruiting the complex and altering gene expression status. The Arp6 subunit of SWR1 has been shown to facilitate binding between certain chromatin domains and nuclear pores, also affecting gene expression (Yoshida et al., 2010). Interestingly, Arp6 can also mediate this binding event independent of its inclusion within the SWR1 complex (Yoshida et al., 2010). Through regulating H2A.Z deposition, Arp6 modulates expression of various genes, as has been demonstrated in several *Arabidopsis* studies. Notably, Arp6 inclusion in SWR1 is crucial for proper plant development and flowering regulation (Choi et al., 2007; Deal et al., 2007). In chicken DT40 cells, Arp6 inclusion in SRCAP promotes rDNA transcription under conditions of high glucose levels by controlling H2A.Z deposition (Kitamura et al., 2015). Moreover, *Arabidopsis* Arp6 has defined roles in repressing certain stress response genes under normal conditions, which is attributed to regulating H2A.Z deposition at certain gene loci (Smith et al., 2010).

Peculiarly, individual SWR1 subunits seem to play contrasting roles in the context of conferring disease resistance to pathogens in *Arabidopsis* (Berriri et al., 2016). While various subunits have been demonstrated to be important in providing biotrophic and necrotrophic pathogen resistance, the presence of the Arp6 subunit actually reduces biotrophic pathogen resistance (Berriri et al., 2016). Unlike mutants in other SWR1 subunits, Arp6 mutation did not hinder effector-triggered immunity following pathogen inoculation (Berriri et al., 2016). Global transcriptome analysis revealed that numerous genes are misregulated in SWR1 subunit mutants, notably in genes conferring disease resistance, consistent with earlier findings (March-Diaz et al., 2008; Berriri et al., 2016). The Arp6 mutant, however, had a transcriptional profile that lacked correlation with other SWR1 subunit mutants, which may explain the mutant’s opposite role in impairing rather than promoting pathogen resistance (Berriri et al., 2016). This finding is in agreement with a prior study showing the Arp6 knockout in yeast results in decreased import of *Agrobacterium* virulence proteins into the cell; moreover, the absence of Arp6 improved degradation of such virulence proteins (Luo et al., 2015). Given that Arp6 is thought to have distinctive roles independent of inclusion in SWR1 (Yoshida et al., 2010), it is possible that Arp6 may counter pathogen resistance in a manner not dependent on SWR1-mediated H2A.Z deposition.

The Arp6 subunit in SWR1 also serves important roles in meiotic recombination in *Arabidopsis*, including carefully regulating expression of the meiotic recombinase DMC1 in megasporocytes while inhibiting its expression in other cell types (Qin et al., 2014). Arp6 inclusion in SWR1 is required for depositing H2A.Z at the *DMC1* gene locus, which likely contributes to this regulation (Qin et al., 2014). Probably as a result of defective control of DMC1 expression and/or other meiotic genes, plants deficient in Arp6 experience a defective female prophase I, manifesting in disorganized chromosomes and unpaired centromeres (Qin et al., 2014). In addition to problems with gametogenesis, Arp6-defective and other SWR1 complex subunit-defective *Arabidopsis* mutants exhibit impaired fertility (Rosa et al., 2013).

The Arp subunits of SWR1 also play key roles in promoting DNA double-strand break repair in somatic cells of certain species (**Figure 2**). In *Arabidopsis*, mutations in several SWR1 subunits, including *Arp6*, sensitize seedlings to a variety of DNA damaging agents, including γ-irradiation, bleomycin, mitomycin C, and hydroxyurea (Rosa et al., 2013). Moreover, DNA lesions accumulate in mutant genomes even in the absence of treatment with DNA damage-inducing agents. Upon exposing Swr1-Ku70 and Swr1-Brca1 double-mutants (defective in non-homologous end-joining and homologous recombination, respectively) to bleomycin, they demonstrated that, unlike Ku70, Brca1 is epistatic with Swr1, indicating that the SWR1 complex acts specifically in homologous recombination (Rosa et al., 2013). Indeed, recombination frequency is drastically diminished in Arp6 and other SWR1 subunit mutants (Rosa et al., 2013). These results are in stark contrast with data in yeast, in which hyper-recombinogenic phenotypes in SWR1 complex mutants have been reported (Kawashima et al., 2007; Horigome et al., 2014). Chromatin remodeling complexes, it seems, contribute differentially to DNA repair processes across evolutionarily distant organisms (**Figure 2**). Future research will need to discern the biological reasons attributable to these differences. Given that different organisms rely on homologous recombination to varying degrees over other double-strand break repair mechanisms, it is conceivable that this factor may contribute to the phenotypes observed in past studies. Alternatively, Arp6 or other SWR1 components may interact with varying factors in different organisms that in turn promote or suppress recombination.

## SWI/SNF (BAF) and RSC (PBAF)

SWI/SNF and RSC are closely related ATP-dependent chromatin remodeling complexes that, through repositioning nucleosomes or evicting histones, are involved in multitudes of cellular processes, ranging from gene activation and repression to cell development and differentiation. In animals, SWI/SNF also has distinctive roles in regulation of brain development (Clapier and Cairns, 2009). In budding yeast, both SWI/SNF and RSC contain Arp7 and Arp9 as an obligate heterodimer (Cairns et al., 1998; Peterson et al., 1998; Szerlong et al., 2003; Schubert et al., 2013), while in human SWI/SNF (BAF) and RSC (PBAF) actin and Arp4 (BAF53) are instead present (Zhao et al., 1998). The Arp7–Arp9 conjugate is recognized by and associated with the HSA domain of the catalytic subunit of the respective complex – Sth1 in yeast RSC, and Snf2 in yeast SWI/SNF (Yang et al., 2007; Szerlong et al., 2008). When in complex with the Snf2 HSA domain, the Arp7–Arp9 heterodimer exists in a compact formation that disfavors filament formation (Lobsiger et al., 2014). This subcomplex in SWI/SNF is sufficient in enabling ATP hydrolysis, as well as nucleosome sliding and histone translocation, though is unable to catalyze the displacement of H2A–H2B dimers (Yang et al., 2007).

Interestingly, the Arp7–Arp9 heterodimer is the only Arp conjugate known to directly associate with each other in a chromatin remodeling complex. This heterodimer may have evolved from the actin-Arp4 heterodimer observed in other chromatin-modifying complexes (Nishimoto et al., 2012). Unlike Arp4 and Arp8 present in INO80, neither Arp7 nor Arp9 exhibit affinity toward histones (Szerlong et al., 2003). ATPase activity is only mildly impaired when Arp7 and Arp9 are depleted in RSC (Szerlong et al., 2003, 2008). Moreover, neither remodeling activity nor RSC assembly relies on the presence of Arp7 and Arp9 in the complex (Yang et al., 2007). Nonetheless, both *Arp7* and *Arp9* are each essential genes in budding yeast, underscoring the distinctive functional importance these Arps must have within the nucleus (Cairns et al., 1998; Peterson et al., 1998). It is possible that these Arp subunits may play a role in stabilizing the activities of other subunits within the complex (Yang et al., 2007). Another such role for Arp7–Arp9 includes its propensity to interact with Nhp6, an architectural transcription factor involved in inducing topological alterations to the chromatin structure, such as DNA bending. RSC promotes binding of Nhp6 to nucleosomal DNA, thereby affecting accessibility to the nucleosome by other downstream factors (Szerlong et al., 2003). Arp7–Arp9 also plays a key role in controlling RSC’s DNA translocation capability, wherein the heterodimer promotes histone sliding and nucleosome ejection (Clapier et al., 2016). Together with Sth1, Arp7–Arp9 enables a particularly efficient DNA translocation rate by increasing the level of translocation per ATP hydrolysis (Clapier et al., 2016). Future study will likely reveal additional critical functions of the Arp7–Arp9 heterodimer in nuclear metabolism.

The human orthologs of SWI/SNF and RSC are BAF and PBAF, respectively. BAF and PBAF each contain an actin and BAF53 (the human homolog of Arp4) subunit (Zhao et al., 1998). Arp4/BAF53 is required for stabilizing the structural integrity of the BAF complex (Nishimoto et al., 2012). Actin and Arp4/BAF53 directly interact with the catalytic ATPase subunit of BAF, BRG1 (Zhao et al., 1998; Nishimoto et al., 2012). Together with Arp4/BAF53, actin promotes BRG1 ATPase activity (Zhao et al., 1998). Treatment with the monomeric actin-inhibiting agent latrunculin-B abrogates BAF’s DNA-dependent ATPase activity, demonstrating the requirement for actin in promoting the function of BRG1 (Zhao et al., 1998). Depletion of Arp4/BAF53 results in degradation of BRG1 in human cell lines (Nishimoto et al., 2012). Arp4/BAF53 also helps target BAF to specific gene promoters, thus regulating expression of a diverse array of BAF-associated processes, including neural differentiation and hemopoietic stem cell proliferation (Lessard et al., 2007; Wu et al., 2007; Yoo et al., 2009; Krasteva et al., 2012). In mice, BRM, which is an alternative catalytic ATPase subunit found in mammalian SWI/SNF complexes that do not contain BRG1, has been shown to interact with a brain-specific isoform of Arp4/BAF53, called ArpN alpha (Kuroda et al., 2002). Expression of this isoform is concurrent with neural differentiation in the brain, suggesting that mammalian SWI/SNF may be implicated in the development of the nervous system (Kuroda et al., 2002). Interestingly, *in vitro* experiments demonstrate that BAF is capable of binding actin filaments in the nucleus, an event stabilized by PIP_2_, a signaling molecule that is known to remove actin-binding proteins from actin (Rando et al., 2002). The complete and functionally active BAF complex is required for this interaction with actin filaments. It is conceivable that binding filamentous actin could serve important functions in anchoring and/or mobilizing the complex throughout the nuclear matrix. It will be interesting to further uncover the functional significance of this interaction with actin filaments, as well as to investigate whether other chromatin-modifying complexes bind actin filaments in a similar manner.

## NuA4 (TIP60)

NuA4 is a yeast histone acetyltransferase that plays key roles in regulating gene transcription, cell cycle control, and certain DNA repair processes following acetylation of histone H4. NuA4 is highly similar in structure and function to the TIP60 histone acetyltransferase complex found in humans (Doyon and Côté, 2004). NuA4/TIP60 contains both actin and Arp4/BAF53, a pair commonly found in several chromatin remodeling complexes, as discussed earlier (Galarneau et al., 2000; Ikura et al., 2000). This actin-Arp4 pair associates with the HSA domain of Eaf1, the catalytic subunit of yeast NuA4 (Szerlong et al., 2008). This interaction is essential for NuA4 complex function and proper acetylation of histone H4 (Steinboeck et al., 2007). Yeast cells lacking Eaf1 exhibit an attenuated growth phenotype, and Eaf1 depletion incurs lethality upon treatment with DNA double-strand break-inducing agents, highlighting the functional importance of this complex (Auger et al., 2008). The ATP-binding pocket of Arp4 is critical to this subunit’s role in modulating the dynamics of the NuA4 complex (Sunada et al., 2005). The binding of ATP allows Arp4 to freely dissociate from the complex, concurrently freeing bound histone substrates (Sunada et al., 2005). Arp4 is required for NuA4 nucleosome binding as well as maintaining stability of the complex (Harata et al., 1999b; Galarneau et al., 2000; Downs et al., 2004).

Arp4 has been shown to recruit the NuA4 complex to DNA double-strand breaks *in vivo*, where NuA4 acetylates histone H4 and helps facilitate the repair process (Bird et al., 2002; Downs et al., 2004) (**Figure 2**). H2A phosphorylation at Ser129, an epigenetic mark indicative of DNA damage in yeast, is recognized by the Arp4 subunit of NuA4, thus enabling its recruitment to the damage site and promoting repair (Downs et al., 2004). NuA4 in turn promotes recruitment of SWI/SNF to double-strand breaks, which in turn further propagates γH2A in the vicinity of the damage site (Bennett and Peterson, 2015) (**Figure 2**). In mammalian cells, TIP60 has key roles in promoting the Fanconi anemia pathway and homologous recombination in response to DNA interstrand crosslinks and double-strand breaks, respectively, in part by promoting expression of genes involved in both pathways, as well as through more direct means (Hejna et al., 2008; Renaud et al., 2016; Su et al., 2017). Whether actin and Arp4/BAF53 are also directly involved in promoting these repair functions of TIP60 remains to be seen. It will be interesting to assess the role actin and Arp4/BAF53 may play in facilitating NuA4/TIP60-mediated DNA repair through combined genetic and biochemical studies.

## Concluding Remarks and Future Perspectives

After a long-lasting and controversial debate on nuclear actin for the past several decades, it is now clear that at least a subset of actin can shuttle in and out of the nucleus in a highly regulated manner. Increasing sets of evidence suggest that nuclear actin plays a pivotal role in regulating different phases of transcription as part of RNA polymerase complexes, as well as a component of chromatin remodelers and histone modifiers. Recent evidence also points to actin’s key roles in other nuclear processes, such as the DNA damage response. The current area of research regarding nuclear actin is now focused on understanding its mechanistic involvement in various nuclear processes and in its different forms. Indeed, the presence of actin-binding proteins and other regulators may render the physical state and conformation of nuclear actin distinct from cytoplasmic actin. Moreover, in the context of chromatin-modifying complexes, nuclear actin relies on its monomeric state, indicating a unique functional role for the protein divergent from canonical actin filaments in the cytoplasm. Combinations of monomeric actin and nuclear Arps dimerize to constitute a key functional module within these complexes, which may facilitate interactions with nucleosomes and in turn promote new conformations that more directly promote catalytic functions of the complex. Exploring the structural and biochemical properties of these actin/Arp modules will likely offer new insights regarding the mechanisms by which these proteins promote chromatin remodeling and histone modification. Moreover, the post-translational modification status of actin remains poorly defined and may play important roles in distinguishing nuclear actin from its cytoplasmic counterpart. Studies in these directions will ultimately uncover the fundamental and highly conserved mechanisms of nuclear actin and Arps.

Despite many recent advances in this rapidly emergent field, many important questions remain unanswered and require attention. How do the different combinations of actin and Arps present in a given chromatin-modifying complex specifically affect function? And does any functional redundancy exist amongst actin and Arps in terms of promoting certain nuclear processes? More broadly, how is actin’s monomeric state controlled within the confines of the nucleus? A more thorough understanding of how actin-binding proteins in the nucleus affect nuclear actin state and function will likely uncover clearer details regarding its regulation. Establishing a more refined notion of how Arps and perhaps nuclear actin cooperate with chromatin remodelers to control and maintain higher-order chromatin structure will also be particularly insightful. While we now are continuing to embark on understanding the role of monomeric actin in the context of chromatin remodelers and histone acetyltransferases, it would also be interesting to investigate the extent to which such complexes associate with actin filaments in the nucleus, as filamentous actin is known to regulate certain nuclear processes such as transcription and DNA double-strand break repair. Furthermore, given the importance of Arps, chromatin remodelers, and histone acetyltransferases in maintaining genomic integrity, it will be insightful to investigate how mutations in actin and Arps contribute to tumorigenesis and various cancers. As we begin to unravel the genetic and biochemical underpinnings associated with nuclear actin and Arps in chromatin-modifying complexes, it is likely that our understanding of how cells regulate processes such as gene transcription, replication, and repair will be greatly enhanced. Given the fundamental importance of actin, the emerging studies of nuclear actin and Arps will likely reveal a new world of actin biology, rivaling that of the cytoplasmic actin.

## Author Contributions

NK-M wrote the manuscript. AK and YZ provided critical input. PK and XS provided expertise, advisement, and critical input.

## Conflict of Interest Statement

The authors declare that the research was conducted in the absence of any commercial or financial relationships that could be construed as a potential conflict of interest.
